# Effects of Interfraction Dose Variations of Target and Organs at Risk on Clinical Outcomes in High Dose Rate Brachytherapy for Cervical Cancer

**DOI:** 10.3390/cancers15194862

**Published:** 2023-10-05

**Authors:** Brien Washington, Dennis Cheek, Denise Fabian, Mahesh Kudrimoti, Damodar Pokhrel, Chi Wang, Cameron Thayer-Freeman, Wei Luo

**Affiliations:** 1 Department of Radiation Medicine, University of Kentucky, 800 Rose Street, Lexington, KY 40536, USA; brien.washington@uky.edu (B.W.); dennis.cheek@uky.edu (D.C.); denise.fabian@uky.edu (D.F.); mahesh.kudrimoti@uky.edu (M.K.); damodar.pokhrel@uky.edu (D.P.); thayerfc@uky.edu (C.T.-F.); 2 Department of Internal Medicine, University of Kentucky, 800 Rose Street, Lexington, KY 40536, USA; chi.wang@uky.edu

**Keywords:** cervical cancer, dose variation, beta distribution, high dose rate brachytherapy, dose response model, utility

## Abstract

**Simple Summary:**

Accurate dose calculation and delivery is critical in treating cancers in radiation therapy. Although high accuracy has been achieved, significant uncertainties or errors still exist in radiation therapy, especially brachytherapy. Various types of uncertainty have been investigated, such as organ filling and organ motion. But interfraction dose variations (IDVs) from the prescribed dose in high dose rate brachytherapy (HDR) resulting from clinical considerations have not been widely discussed. Understanding what IDVs look like and whether or not they would affect clinical outcomes is important to improve the effectiveness of HDR brachytherapy and quality of cervical cancer treatment. We found IVDs followed a left-skewed distribution in our previous study, and we continued to discuss their potential effect on clinical outcomes in this project. In this study, we found that IDVs would reduce tumor control probability and increase treatment failure rate, but a dose escalation could be a remedy for such an effect.

**Abstract:**

Meeting dose prescription is critical to control tumors in radiation therapy. Interfraction dose variations (IDVs) from the prescribed dose in high dose rate brachytherapy (HDR) would cause the target dose to deviate from the prescription but their clinical effect has not been widely discussed in the literature. Our previous study found that IDVs followed a left-skewed distribution. The clinical effect of the IDVs in 100 cervical cancer HDR patients will be addressed in this paper. An in-house Monte Carlo (MC) program was used to simulate clinical outcomes by convolving published tumor dose response curves with IDV distributions. The optimal dose and probability of risk-free local control (RFLC) were calculated using the utility model. The IDVs were well-fitted by the left-skewed Beta distribution, which caused a 3.99% decrease in local control probability and a 1.80% increase in treatment failure. Utility with respect to IDV uncertainty increased the RFLC probability by 6.70% and predicted an optimal dose range of 83 Gy–91 Gy EQD2. It was also found that a 10 Gy dose escalation would not affect toxicity. In conclusion, HRCTV IDV uncertainty reduced LC probabilities and increased treatment failure rates. A dose escalation may help mitigate such effects.

## 1. Introduction

Brachytherapy procedures are subject to varying levels of uncertainties at every step of the brachytherapy process [1,2,3]. Guidance on handling specific uncertainties was provided by the American Association of Physicists in Medicine Task Group 138 (AAPM TG-138), but clinical uncertainties were left for clinicians to investigate. Clinical uncertainties are uncertainties that clinicians have some control over. They include, but are not limited to, structure contouring and motion [4,5,6,7,8,9,10,11,12,13,14]. Interfraction variation in position of the uterus during external beam radiation therapy and high dose rate (HDR) brachytherapy have been reported in the literature [15,16,17]. It is generally accepted that structure contouring and motion are the dominant forms of clinical uncertainties in HDR brachytherapy for cervical cancer [18]. However, other forms of clinical uncertainties exist that can affect dosimetry and clinical outcomes.

Dose to a target or dose to an organ at risk (OAR) can vary from the prescribed dose for the target or the expected dose for the OAR. Interfraction dose variations (IDVs) from the prescribed dose are considered a form of uncertainty (IDV uncertainty) in adaptive brachytherapy procedures like HDR. Adaptive HDR brachytherapy procedures are prone to IDV uncertainty. Both Hellebust et al. [6] and Nesvacil et al. [19] quantified a 10% uncertainty for the dose delivered to 90% ($D_{90}$) of the high-risk clinical target volume (HRCTV) as a function of interobserver contour variability and structure motion, respectively, in HDR brachytherapy for cervical cancer. IDVs do not dominate the brachytherapy uncertainty budget but can still influence dosimetry and clinical outcomes.

The clinical effect of uncertainties can be investigated using biological modeling [20,21]. Tumor control probability (TCP) and normal tissue complication probability (NTCP) are common biological models used in radiotherapy. Nesvacil et al. [21] developed a TCP Monte Carlo (MC) simulation model to hypothesize the clinical effect that a 10% IDV uncertainty, described as a Gaussian-random variable, has on local control (LC). They found that IDV uncertainty has a minor effect on LC if one assumes IDV uncertainty is a Gaussian-random variable. In our previous study on adaptive HDR brachytherapy dosimetry, we found evidence suggesting IDV uncertainty may not be considered a Gaussian-random variable [22]. Instead, IDV uncertainty in cervical cancer HDR brachytherapy was a non-normal, left-skewed distribution yielding a 30.0% probability of under-dosing the HRCTV. In this study, we aim to quantify the clinical effect of IDV uncertainty in HDR brachytherapy for cervical cancer using IDV uncertainty distributions derived from dosimetric data and biological modeling.

## 2. Methods and Materials

### 2.1. Calculating IDV and Determining IDV Uncertainty Distributions

In this study, IDV uncertainty was defined as the percent difference between the delivered equivalent dose in a 2 Gy fraction ($EQD_{2}$) and the prescribed or expected $EQD_{2}$ (Equation (1)). In our previous study, IDV uncertainty was defined as the mean dose variation from the prescribed dose over a course of HDR adaptive brachytherapy [22]. Using the $EQD_{2}$ to calculate IDV uncertainty incorporates interfraction dose fluctuations by the definition of $EQD_{2}$, and is more appropriate to use with TCP and NTCP models since they are derived using $EQD_{2}$. The HRCTV, rectum, bladder, and sigmoid were the structures evaluated in this study. HRCTV IDVs were calculated with the delivered $D_{90}$ $EQD_{2}$ and the prescribed $EQD_{2}$ (α/β = 10 Gy). The corresponding OAR IDVs were calculated with the dose delivered to the most exposed 2 $cm^{3}$ ($D_{2cc}$) $EQD_{2}$ and the expected OAR $EQD_{2}$ derived from the prescription dose (α/β = 3 Gy). One-hundred cervical cancer patients (FIGO stage IB-IVB) treated with HDR T&O adaptive brachytherapy at our institution from 2018–2020 had their IDVs calculated. The HDR $EQD_{2}$ prescriptions ranged from 18.8–40 Gy (α/β = 10 Gy).

(1) $$IDV\;Uncertainty\;(\%)=\frac{Delivered\;EQD_{2}-Prescription\;or\;expected\;EQD_{2}}{Prescription\;or\;expected\;EQD_{2}}\;\cdot \;100$$

Determining the best-fit HRCTV IDV distribution was described in detail in our previous study [22]. In brief, Python 3.7′s DistFit package was used to fit the IDV distributions to 89 different probability density functions (PDF) with limited dependence on distribution bin width. The fits were ranked using residual sum of square (RSS) scoring. The DistFit analysis provided the RSS score, shaping parameters, plotting location, and the scale of the PDF. The Anderson–Darling test was used to determine if the IDV uncertainty distribution was normally distributed or not. The null hypothesis for the Anderson–Darling test is that the distribution fits the data, so *p*-values < 0.05 indicate the distribution does not fit the data. Quantile–quantile (Q–Q) plots were used to visualize the Anderson–Darling test’s results.

Two HRCTV IDV uncertainty distributions were examined in this study: the best fit distribution and a standard normal distribution (SND). The best fit distribution was determined from the RSS scores, and the SND had a mean IDV of 0.00% and a standard deviation equal to the standard deviation of the raw IDV data. The best-fit distribution represented IDV uncertainty as a left-skewed-random variable, while the SND represented it as a Gaussian-random variable. The Anderson–Darling test was applied to the best fit distribution and the SND.

The uncertainty distributions of OAR IDVs were calculated with respect to OAR IDV correlations with HRCTV IDVs [22]. If the OAR IDVs were not correlated via linear regression (*p* < 0.05) to HRCTV IDVs, then that structure was not used in the simulation model.

### 2.2. Reference Dose Response Curves

Published cervical cancer radiotherapy TCP and NTCP curves were used as a reference in this study [23,24,25]. The reference TCP and NTCP curves used Logistic (Equation (2)) and Probit (Equation (3)) regression techniques to derive their dose response curves:

(2) $$R=\frac{1}{1+e^{4\gamma (1-\frac{D}{D_{50}})}}\;\;,$$

(3) $$R=0.5+0.5\mathrm{erf}\left(\frac{t}{\sqrt{2}}\right),\;\;t=\frac{D-D_{50}}{{\left(\gamma \sqrt{2\pi }\right)}^{-1}D_{50}}\;\;.$$

where R is the dose response probability, *γ* is the steepness parameter, and $D_{50}$ is the dose of 50% response.

Four TCP curves derived from three-year LC rates of the HRCTV $D_{90}$ dose were used: the TCP curve derived by Tanderup et al. [23] from the retroEMBRACE data (TCP1, N = 488, $D_{50}$ = 36.0 Gy, *γ* = 0.47), and the three-tumor size specific TCP curves derived by Dimopoulos et al. [24] (TCP2A-C). The three TCP2 groups were: gross tumor volume (GTV) diameters > 2 cm (TCP2A, N = 141, $D_{50}$ = 45.0 Gy, *γ* = 0.60), GTV diameters > 5 cm (TCP2B, N = 77, $D_{50}$ = 61.0 Gy, *γ* = 1.10) at diagnosis, and HRCTV diameters > 5 cm post-EBRT (TCP2C, N = 31, $D_{50}$ = 68.0 Gy, *γ* = 2.00). In this study, TCP1 and TCP2A served as an aggregate for cervical cancer radiotherapy, TCP2B represented large tumors, and TCP2C represented large tumors having a poor EBRT response prior to brachytherapy. The reference NTCP curve was derived by Georg et al. [25]. (N = 141 patients, $D_{50}$ = 110 Gy, *γ* = 2.00) from late effects of the rectum and bladder $D_{2\;cc}$.

### 2.3. Monte Carlo Simulation and Convolution

The effect of IDV uncertainty on clinical outcomes was estimated by convolving reference dose response curves with a respective IDV uncertainty distribution (Equation (4)):

(4) $$R^{\prime }(D)=R\left(D^{\prime }\right)\;⨂\;IDV(D-D^{\prime })\;.$$

where $R^{\prime }(D)$ is the convolved dose response curve, $R\left(D^{\prime }\right)$ is the reference dose response curve, ⨂ is the convolution operator, and $IDV(D-D^{\prime })\;$ is the IDV uncertainty distribution. An in-house Monte Carlo (MC) Simulation Convolution model was developed using Python 3.7 to convolve the dose response curves.

The reference dose response curves were convolved with the IDV uncertainty distributions in the range of clinical interest (RoCI). The RoCI represented the total dose of cervical cancer radiotherapy (70–100 Gy $EQD_{2}$), implying HDR prescription of 25–55 Gy plus 45 Gy of EBRT in 25 fractions. HDR treatments were simulated 10,000 times at each HDR prescription in the RoCI by sampling IDVs from IDV uncertainty distributions (IDV(D)) and applying them to the HDR prescription. The simulated HDR treatments were summed with 45 Gy in 25-fraction EBRT $EQD_{2}$ to simulate cumulative delivery, D. This dose has a corresponding probability on the reference dose response curves, $R\left(D\right)$. A random number between 0 and 1 was applied to each treatment and compared to the TCP from the reference model at each corresponding prescription dose. This was used to generate a binary clinical outcome for each simulated treatment. An in-house logistic regression algorithm was used to generate new TCP curves for the simulated data using maximum-likelihood estimation.

Sampling of OAR IDVs was conducted with respect to OAR and HRCTV IDV correlations. Each sampled IDV from the HRCTV IDV uncertainty distribution, $IDV(D-D^{\prime })$, was inputted in the linear regression equation. The output OAR IDV was selected by randomly sampling at the derived value from a normal distribution with a mean value equal to the derived value from the equation and standard deviation equal to the residuals from the linear regression. This accounted for OAR and HRCTV IDV correlations in the simulation.

### 2.4. Determination of Treatment Response and Treatment Failure Rate

Treatment failures are generally defined as local recurrences or local failures in cervical cancer radiotherapy clinical outcome studies [24]. Treatment responses for all simulated treatments were determined via probabilities from the reference dose response curves and an event simulator. The probabilities obtained from the reference dose response curves only provided the likelihood of a treatment response; they did not simulate a treatment response. An event simulator was used to simulate treatment responses with respect to the likelihood of observing that response. The event simulator dichotomized the data: 1 for treatment response, and 0 for no treatment response.

The 3-year treatment failure rate was calculated from the dichotomized data. The percentage of non-treatment responses served as the treatment failure rate for the HRCTV. The 3-year treatment failure rate was also calculated without IDV uncertainty to serve as a control variable. This process was repeated 30 times to satisfy the central limit theorem. The mean treatment failure rates as a function of IDV uncertainty distribution sampling were calculated from these data.

### 2.5. Utility Analysis

The probability of risk-free local control (RFLC) can be computed from TCP and NTCP curves. This is known as the utility of a treatment and was proposed by Schultheiss et al. [26] and used by Boyer et al. [20]. Analytically, utility is defined as:

(5) $$U\left(D\right)=TCP\left(D\right)\left(1-NTCP\left(D\right)\right).$$

where $U\left(D\right)$ is the utility function, and $TCP\left(D\right)$ and $NTCP\left(D\right)$ are the dose response curves. Subtracting the NTCP by one gives the probability of not having a complication. Thus, multiplying this probability by the TCP gives the probability of RFLC. The dose with maximum RFLC probability is the predicted optimal dose. The utility of the simulated dose response curves, $R^{\prime }$, was calculated for every $R^{\prime }$ in this study and was evaluated against the utility of the reference dose response curves.

### 2.6. Statistical Analysis

Dose response probability differences in the RoCI were also evaluated. Dose response probabilities greater than 1% were of interest in this study [21]. The 85 Gy probability differences for the TCPs were deemed most important per literature recommendations [23,24,26]. Standard deviation counts from the control treatment failure rate were used for treatment failure rate analysis. RFLC probability and optimal dose prediction differences were evaluated for utility analysis.

### 2.7. Accuracy Testing of the Model

The model was tested by running a simulation on the reference dose response curves without any IDV uncertainty applied. The expected dose response curve and failure rate are known from the reference dose response curves. As mentioned prior, these served as the control variable for the treatment failure rate analyses. Deviations from the reference curve were the model’s statistical uncertainty.

## 3. Results

### 3.1. HRCTV and OAR IDV Uncertainty

**Figure 1:**
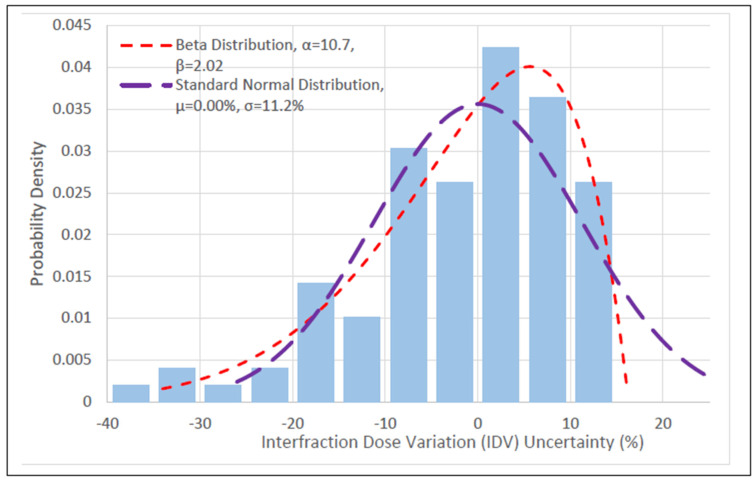
Fitted distributions for HRCTV *D*90 determined from the data driven analysis (RSS score). The HRCTV *D*90 variations from prescriptions are on the x-axis and the corresponding probability density is on the y-axis.

**Figure 2:**
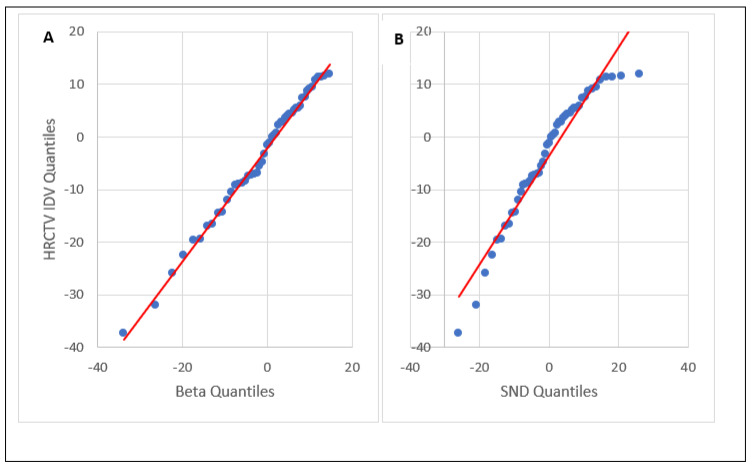
Q–Q plots for the Anderson–Darling test: (**A**) Beta; (**B**) SND. Blue points represent each of individual data points, and the red line represents the line of best fit.

The mean HDR prescription and delivered $EQD_{2}$ were 29.4 ± 7.44 Gy and 29.1 ± 8.17 Gy, respectively. These yielded a mean IDV of −1.53 ± 11.2%. The mean HRCTV was 53.0 ± 34.3 $cm^{3}$. The Beta distribution, generalized extreme value (GEV) distribution, and SND were used to fit the IDV data. Table 1 summarizes the values of the fitting parameters of the distributions. The left-skewed Beta distribution was the best-fit HRCTV IDV uncertainty distribution with an RSS score = $1.16\;\cdot 10^{-2}$. The left-skewed generalized extreme value (GEV) distribution was the second-best-fit distribution with an RSS score = $1.37\;\cdot 10^{-2}$ and an Anderson–Darling *p*-value > 0.251, consistent with our previous study’s findings [22]. The Beta distribution’s Anderson–Darling *p*-value was estimated to be > 0.251 based on the GEV. The SND was an inferior fit to the Beta and GEV distributions with an Anderson–Darling *p*-value < 0.0002. This suggests that HRCTV IDV uncertainty does not follow a normal distribution. As the SND was assumed by other studies, it was used for comparison in this study. The HRCTV IDV data, and Beta and SND distributions used for IDV sampling are shown in Figure 1. The quantile–quantile (Q–Q) plots for the Anderson–Darling test are compared between Beta and SND distributions in Figure 2.

**Figure 3:**
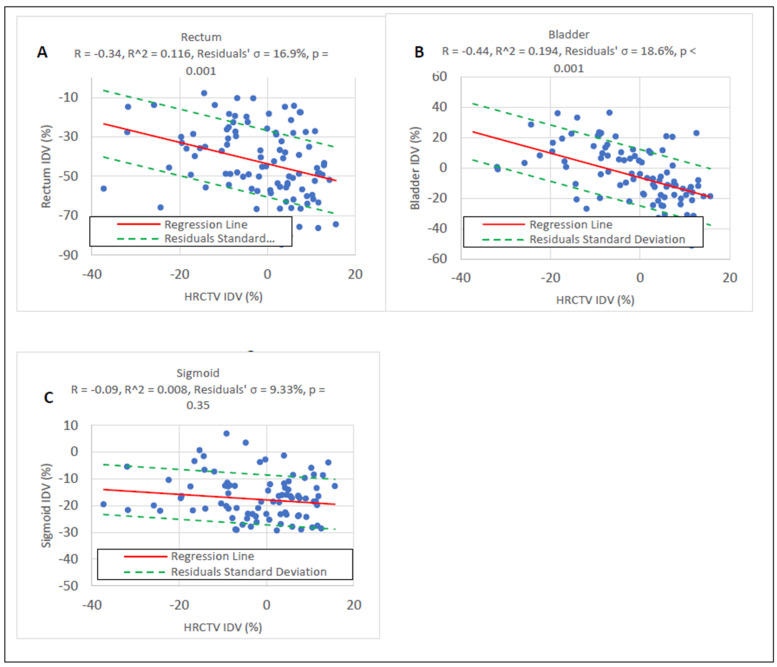
(**A**–**C**) Linear regression of the OAR IDV data with the HRCTV IDV data. OAR IDV uncertainty sampling was conducted using the HRCTV IDV selected from the Monte Carlo simulation and the equation from the regression models of statistically significant correlations.

As with our previous study [22], rectum and bladder IDVs were moderately correlated with HRCTV IDVs and sigmoid IDVs were not correlated to HRCTV IDVs based on the linear regression model. This is shown in Figure 3. Thus, only the rectum and bladder were used for IDV uncertainty sampling in the clinical outcome simulations to account for IDV uncertainty correlations.

### 3.2. Effect of IDV Uncertainty on TCP and NTCP

**Figure 4:**
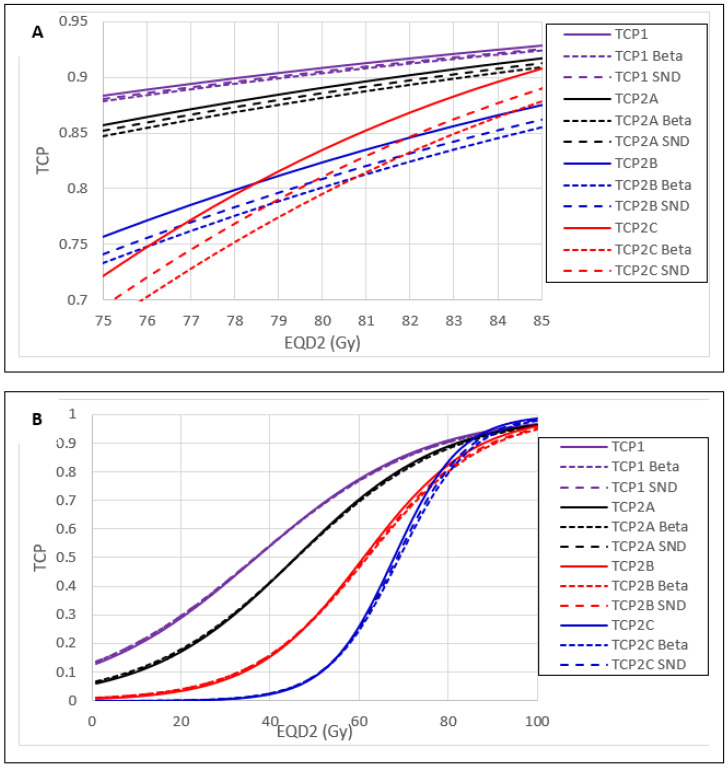
Comparison of TCP curves between R’s and Rs. (**A**). TCP curves over the RoCI. (**B**). TCP curves from 1–100 Gy EQD2. TCP1 and TCP2B had smaller local control (LC) probability differences when compared to TCP2B and TCP2C.

Figure 4A displays $R^{\prime }$ versus R LC probability differences over the RoCI, and Figure 4B displays these from 1–100 Gy EQD2. Beta IDV-sampling had larger LC probability reductions than SND-IDV sampling. TCP1 had no LC probability reductions beyond −1%. The HRCTV size-dependent TCP2B and TCP2C were sensitive to all three IDV uncertainty distributions. Beta IDV sampling reduced the LC probability by 2.02% and 2.98% at 85 Gy for TCP2B and TCP2C, respectively. SND IDV sampling reduced the LC probability by 1.35% and 1.82% at 85 Gy for TCP2B and TCP2C, respectively.

The model-generated $R^{\prime }$ curves had up to a −3.99% LC probability difference from the reference TCP curves. Up to a −1.56% difference in TCP was found between the Beta and Normal IDV affected responses. At 85 Gy EQD2, one of most used prescribed doses, TCP2B, showed sensitivity to the Beta IDV uncertainty distribution (−2.02%), and TCP2C showed sensitivity to both IDV uncertainty distributions (−2.98% and −1.82% for the Beta and normal-sampled $R^{\prime }$ curves, respectively). The TCP1 and TCP2A were robust to IDV uncertainty. Figure 4 displays the LC differences from the reference TCP curves.

**Figure 5:**
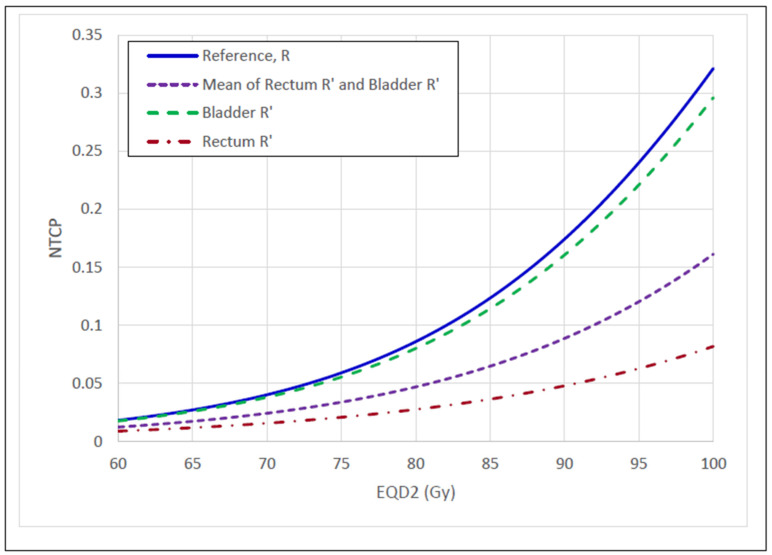
Comparison of NTCP curves between R’ and R. The rectum, bladder, and sigmoid R’ NTCP curves were derived from convolving the reference NTCP curves with their respective IDV uncertainty distribution. The OAR R’ NTCP curve is the mean of the rectum, bladder, and sigmoid R’ NTCP curves. A 10 Gy HRCTV dose escalation from 80–90 Gy was observed from the reference NTCP curve and OAR R’ NTCP curve.

The rectum $R^{\prime }$ reduced the morbidity probability by 3.80% at the recommended 75 Gy $EQD_{2}$ rectal-tolerance dose, and further reduced the morbidity probability throughout the RoCI. The bladder $R^{\prime }$ only reduced the morbidity probability at higher doses, with a 1.36% reduction at the recommended 90 Gy $EQD_{2}$ tolerance dose. Combining the rectum and bladder $R^{\prime }$ curves resulted in morbidity probability reductions of 2.5% and 8.6% at 75 and 90 Gy, respectively. These morbidity probability reductions hypothetically allow for an approximate 10 Gy HRCTV dose escalation from 80 Gy to 90 Gy (Figure 5).

### 3.3. Utility Analysis

**Figure 6:**
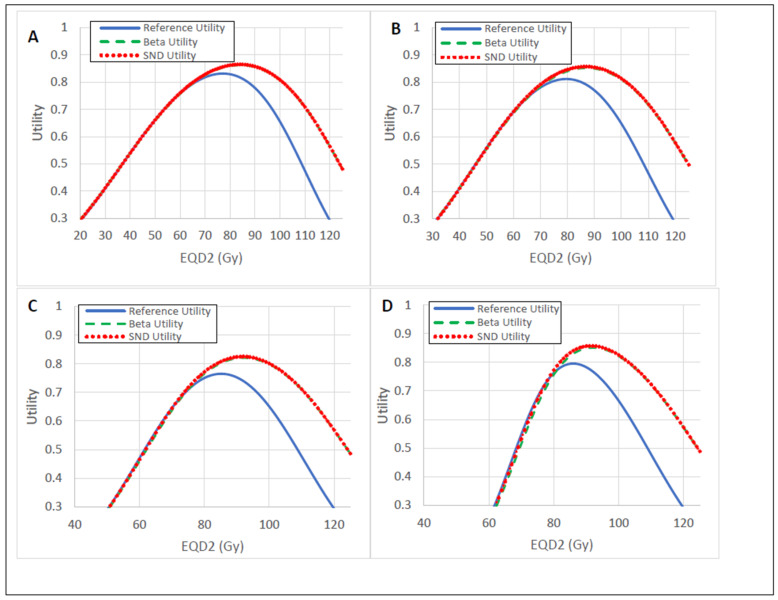
Utility curves for R’ and R: (**A**) TCP1; (**B**) TCP2A; (**C**) TCP2B; (**D**) TCP2C. IDV uncertainty increased the optimal dose prediction and the probability of risk-free local control (RFLC).

The Beta IDV-sampled utility yielded an optimal dose and RFLC probability range of 84–92 Gy and 82.1–86.3%, respectively (Table 2 and Figure 6), for each respective TCP curve. The optimal doses corresponded to the maximum utility values. This resulted in an optimal dose increase of as much as 7 Gy, and a RFLC probability increase of as much as 5.6% for the Beta IDV-sampled utility. Similar results were observed for SND IDV-sampled utility.

### 3.4. Treatment Failure Rate

**Figure 7:**
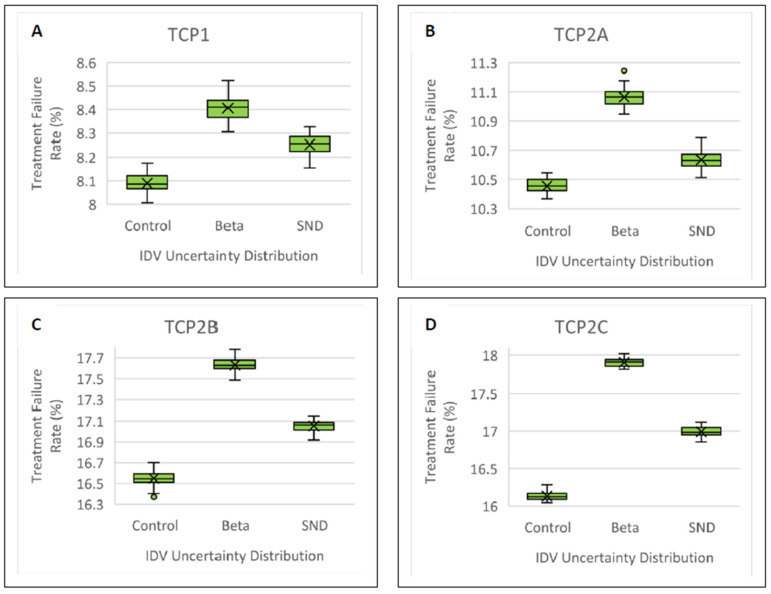
Comparison of treatment failure rate predictions and analysis between the Beta, SND, and control IDV distributions for different tumor sizes: (**A**), TCP1; (**B**), TCP2A; (**C**), TCP2B; and (**D**), TCP2C. The Beta was statistically different from the SND and control in every case.

The treatment failure rate increased for the Beta IDV uncertainty distribution versus the control treatment failure rate (*p* < 0.001). Beta IDV sampling increased the treatment failure rate by as much as 1.80% (35.7σ) for TCP2C (Figure 7 and Table 3). A 1.00% (14.3σ) increase in treatment failure rate was also observed for Beta IDV-sampling for TCP2B. As with the convolution results, this implies increased sensitivity to IDV uncertainty for larger HRCTVs. The Beta IDV-sampled treatment failure rates were statistically different from the SND-sampled treatment failure rates (Table 4).

## 4. Discussion

Multiple clinical outcome simulations under the influence of interfraction dose variations (IDV) from the prescribed or expected dose (IDV uncertainty) were successfully conducted. We have doubled our sample size and found that IDV uncertainty is still best described as a non-normal, left-skewed distribution. More importantly, the left-skewed distribution is indicative of under-dosing the high-risk clinical target volume (HRCTV). We have shown that IDV uncertainty can result in higher treatment failure rates, reduced local control (LC) probabilities, and reduced morbidity probabilities. We have also shown that HRCTV and OAR IDV uncertainties increase the probability of risk-free local control (RFLC) and predict an optimal dose consistent with recommended cervical cancer radiotherapy prescriptions via the utility model.

Interfraction dose variation (IDV) uncertainty in adaptive procedures is not a dominant portion of the brachytherapy uncertainty budget, however, it is relevant for clinical investigation. Nesvacil et al. found that IDV uncertainty has a minor effect on clinical outcomes if one assumes IDV uncertainty is a Gaussian-random variable, but systematic uncertainties can reduce the LC probability by as much as 5% at 85 Gy $EQD_{2}$ [21]. Although we did not investigate systematic uncertainties in this study, we have found that IDV uncertainty can negatively affect clinical outcomes when accurate IDV uncertainty distributions are used in clinical outcome simulations. The left-skewed Beta distribution was fitted to clinical IDV data to accurately quantify IDV uncertainty. This distribution incorporated the increased likelihoods of HRCTV under-dosing with their respective skewness, and mean IDV values being less than 0.00%. The Beta IDV uncertainty distributions resulted in reduced LC probabilities and increased treatment failure rates when compared to the control (without uncertainty) and the SND IDV uncertainty distribution. This phenomenon was most profound for larger HRCTVs. Recall that reference curves TCP2B and TCP2C represented larger target volumes [21]. We observed the largest differences in LC probability (−2.02% and −3.99%) and treatment failure rate (1.0% and 1.8%) for TCP2B and TCP2C, respectively. This suggests increased sensitivity to IDV uncertainty for patients with larger tumors. Large target volumes are not a new problem in brachytherapy, but we have shown their dosimetric and clinical implications with respect to IDV uncertainty. There are ways to mitigate IDV-uncertainty related coverage loss: vaginal packing or recto-vaginal spacers can increase the distance between the HRCTV and rectum, and interstitial needles can increase dose coverage [27,28,29]. Vaginal packing is a standard of care when applicable, but recto-vaginal spacers are not available to all clinics. Furthermore, interstitial procedures are recommended to have only one or two implantations to reduce morbidity and patients may need to be admitted as inpatients [29]. Testing these methods was beyond the scope of this study and is recommended for future studies.

Limiting OAR dose is often the secondary objective in radiotherapy. We have shown that OAR IDV uncertainty drastically reduces the complication probability and hypothetically allows dose escalation to the HRCTV (Figure 5 and Figure 6). This result is consistent with what has been found in other simulations and clinical studies [21,27,28,29]. It may not be intuitive that OAR and HRCTV IDV uncertainties can be combined to predict dose escalation to the HRCTV; however, our utility calculation predicted just that. We accounted for the correlations between OAR IDV uncertainties with HRCTV IDV uncertainty to predict both dose escalation and an increase in RFLC probability. The optimal dose range prediction is consistent with clinical prescription dose recommendations [23,24,30]. It is not lost upon the authors that the utility results predominantly come from OAR IDV uncertainty. Therefore, we recommend a future study on the actual clinical outcomes with respect to HRCTV and OAR IDV uncertainties to mitigate any statistical uncertainties that may exist from this prediction. A prospective or retrospective study with different applicators yielding different IDV uncertainties should suffice.

Our results show IDV uncertainty has a noticeable clinical effect, but limited consideration of patient tumor volume for the IDV uncertainty distribution calculation is a limitation of this study, for that could affect the dosimetry and the simulated clinical outcomes. Logistic and Probit dose response curves have inherent uncertainties [31]. Thus, these inherent uncertainties and inaccuracies are also considered a limitation in this study. There is an emphasis in the literature to use additional parameters to evaluate dosimetry and predict clinical outcomes [12,14,23,24,29,32,33,34]. The inclusion of multiple parameters such as volume, tumor size, tumor asymmetry, and dose location will all be of interest for future research. Interfraction contour variability and CT delineation uncertainty was not considered in this study but will be discussed in future research, too.

## 5. Conclusions

Interfraction dose variations (IDV) from the prescribed dose (IDV uncertainty) in HDR brachytherapy for cervical cancer has non-negligible effects on clinical outcomes. HRCTV IDV uncertainty was best described as a left-skewed distribution. Accurate HRCTV IDV uncertainty distributions increase the treatment failure rate and reduce local control probability. This phenomenon was more potent for large tumors. Consideration of OAR IDV uncertainty can reduce the complication probability and theoretically escalate dose to the HRCTV. Evaluation of the utility model under the influence of HRCTV and OAR IDV uncertainties also increased the optimal dose prediction and increased the risk-free local control probability. The optimal dose predictions with respect to HRCTV and OAR IDV uncertainties are consistent with known clinical prescription dose recommendations.

## Figures and Tables

**Table 1:** Distribution statistics for the target dose variation (DV) uncertainty distributions used in this study. The Double Weibull, Beta, and GEV distributions represented the rectum, bladder, and sigmoid, respectively.

Distribution	RSS Score	Mean IDV ± Std (%)	*p*
Beta	${1.16\;\cdot 10}^{-2}$	−1.53 ± 11.0	>0.251 *
Generalized Extreme Value **	${1.37\;\cdot 10}^{-2}$	−1.54 ± 11.1	>0.251
Standard Normal	${5.89\;\cdot 10}^{-2}$	0.00 ± 11.2	<0.0002

* The Beta distribution Anderson–Darling *p*-value was estimated from the GEV *p*-value calculation. ** The GEV distribution was not used for IDV sampling in this study.

**Table 2:** Comparison of optimal doses (EQD_2_) and corresponding utilities (U) calculated with IDV and without IDV (reference).

	Reference	Beta	SND
TCP1	77.0 Gy, 83.2%	84.0 Gy, 86.3%	84.0 Gy, 86.6%
TCP2A	80.0 Gy, 81.2%	87.0 Gy, 85.2%	87.0 Gy, 85.8%
TCP2B	85.0 Gy, 76.5%	92.0 Gy, 82.1%	92.0 Gy, 82.5%
TCP2C	86.0 Gy, 79.5%	92.0 Gy, 85.1%	91.0 Gy, 85.7%

**Table 3:** Statistical analysis of predicted treatment failure rates for IDV sampling from the Beta, SND, and control. The Beta was statistically different from the SND and control.

Reference TCP	Control	Beta		SND	
	Treatment Failure Rate (%)	Treatment Failure Rate (%)	σ Away from Control	Treatment Failure Rate (%)	σ Away from Control
TCP1	8.10 ± 0.05	8.41 ± 0.06	7.03	8.25 ± 0.05	3.40
TCP2A	10.45 ± 0.05	11.0 ± 0.05	11.0	10.7 ± 0.06	5.00
TCP2B	16.6 ± 0.07	17.6 ± 0.07	14.3	17.0 ± 0.06	5.71
TCP2C	16.1 ± 0.06	17.9 ± 0.08	35.7	17.0 ± 0.07	17.8

**Table 4:** *T*-test *p*-values for the Beta treatment failure rates against the SND treatment failure rates. The Beta was statistically different from the SND and control.

	Beta and SND
TCP1	<0.001
TCP2A	<0.001
TCP2B	<0.001
TCP2C	<0.001

## Data Availability

The data presented in this study are available in this article.
